# Global burden of type 2 diabetes mellitus from 1990 to 2021, with projections of prevalence to 2044: a systematic analysis across SDI levels for the global burden of disease study 2021

**DOI:** 10.3389/fendo.2024.1501690

**Published:** 2024-11-08

**Authors:** Ke-Jie He, Haitao Wang, Jianguang Xu, Guoyu Gong, Xu Liu, Huiting Guan

**Affiliations:** ^1^ The Quzhou Affiliated Hospital of Wenzhou Medical University, Quzhou People’s Hospital, Quzhou, Zhejiang, China; ^2^ The School of Clinical Medical Sciences, Southwest Medical University, Luzhou, Sichuan, China; ^3^ School of Medicine, Xiamen University, Xiamen, China; ^4^ Department of Neurology, The Second Affiliated Hospital of Xuzhou Medical University, Xuzhou, China; ^5^ Shenzhen Bao'an Chinese Medicine Hospital, Guangzhou University of Chinese Medicine, Shenzhen, China

**Keywords:** type 2 diabetes mellitus, disability-adjusted life years, mortality, age-period-cohort analysis, epidemiological transition, socio-demographic index

## Abstract

**Background:**

We aimed to assess temporal trends in type 2 diabetes mellitus (T2DM)-related deaths and disability-adjusted life years (DALYs) at global and cross-social demographic index (SDI) levels, using data from the Global Burden of Disease (GBD) in 2021.

**Methods:**

We used geospatial mapping to visualize the global distribution of T2DM-related mortality and DALYs in 2021. Joinpoint regression assessed annual and average percent changes in DALYs and deaths from 1990 to 2021 across SDI regions. Age-period-cohort modeling examined the effects of age, period, and cohort on trends. Decomposition analysis evaluated the impact of population growth, aging, and epidemiological changes on DALY trends. A stratified projection forecasted future T2DM burden by age and sex from 2020 to 2044.

**Results:**

T2DM-related mortality and DALYs were highest in low-SDI regions. Globally, T2DM-related deaths and DALYs have increased, with the most rapid rise in low and low-middle SDI regions, driven by population growth and epidemiological shifts. High-SDI countries showed a slower increase in DALYs, influenced more by aging. Age-period-cohort analysis indicated higher DALY rates in later birth cohorts and recent periods, especially in high-SDI regions. Future projections show a significant increase in the 70-74 age group and a gradual rise in other age groups.

**Conclusion:**

The burden of T2DM is projected to continue increasing, especially in low-SDI and low-middle SDI regions, where population growth and epidemiological shifts are the main contributors. This underscores the need for targeted, region-specific healthcare policies, preventive strategies, and age-specific interventions to address the increasing T2DM burden globally.

## 1 Introduction

Type 2 diabetes mellitus (T2DM) has emerged as one of the most prevalent chronic diseases worldwide, with significant implications for global public health (1). Characterized by insulin resistance (2) and relative insulin deficiency (3), T2DM leads to numerous long-term complications, including cardiovascular disease (4), kidney failure (5), neuropathy (6), and retinopathy (7). The global rise in T2DM incidence has been closely associated with rapid lifestyle changes, population growth, and increased longevity, particularly in developing countries undergoing urbanization and industrialization (8, 9).

The burden of T2DM is often measured through mortality and disability-adjusted life years (DALYs) (10), which reflect both premature mortality and the years lived with disability (11). DALYs provide a comprehensive metric to assess the total impact of T2DM, making them critical for the formulation of healthcare policies and intervention strategies. Global trends indicate substantial variations in T2DM-related mortality and DALYs, largely driven by socio-economic and demographic factors (10). The socio-demographic index (SDI), which combines income, education, and fertility rates, has become a key framework for understanding disease burden across countries at different stages of development (12, 13).

In the context of global health transitions, there has been a marked shift from infectious diseases to non-communicable diseases (NCDs) like T2DM, especially as populations age (14). This epidemiological transition is altering the landscape of global health, with non-communicable diseases becoming the leading cause of morbidity and mortality (15). Despite the increasing recognition of this transition, comprehensive assessments of how population dynamics, aging, and epidemiological changes influence T2DM-related deaths and DALYs across different SDI levels remain limited. Understanding the relative contributions of age, period, and birth cohort effects to these trends is critical for projecting future disease burden and shaping effective public health interventions.

To address this gap, we conducted a detailed analysis of the temporal trends in T2DM-related DALYs and mortality from 1990 to 2021, with a focus on global and regional patterns across different SDI levels. We applied advanced epidemiological techniques, including Joinpoint regression analysis to evaluate changes in T2DM burden over time, and age-period-cohort modeling to disentangle the effects of aging, period, and cohort dynamics. Additionally, a decomposition analysis was performed to quantify the contributions of population growth, aging, and epidemiological shifts to the observed trends in DALYs.

This comprehensive approach allows us to identify the key drivers of the rising global T2DM burden, offering insights that are crucial for the development of tailored healthcare policies. By understanding the differential impact of population aging in high-SDI regions and rapid population growth in low- and middle-SDI regions, our study provides a framework for region-specific interventions. These interventions can help mitigate the future burden of T2DM and its associated complications, especially in the most vulnerable populations.

## 2 Materials and methods

### 2.1 Overview

In this study, we utilized data from the Global Burden of Disease (GBD) study, managed by the Institute for Health Metrics and Evaluation (IHME). The GBD database is an annually updated global health database that encompasses a wide array of diseases and associated health metrics (10, 16).

### 2.2 Data sources

Our primary data on T2DM-related mortality and DALYs were extracted from the Global Burden of Disease Collaborative Network and the Global Burden of Disease Study 2021 results. These sources provide comprehensive, age-standardized rates of T2DM mortality and DALYs, which are cataloged by age, sex, year, and SDI region, and are publicly accessible through IHME’s various visualization tools (https://vizhub.healthdata.org/gbd-results/). We specifically analyzed data spanning from 1990 to 2021, focusing on global trends and projections up to 2044, which are also derived from these datasets. To provide a more thorough explanation of the steps we took to ensure data accuracy and reliability, we have included additional details in **Supplementary Material 1**.

### 2.3 Analytical methods

To evaluate the temporal trends and forecast the future burden of T2DM, we employed several statistical and modeling techniques:

#### 2.3.1 Joinpoint regression analysis

This method was used to analyze changes in T2DM DALYs and mortality rates over time. It identifies points where significant changes in trend occur and estimates the annual percent change and average annual percent change (AAPC).

#### 2.3.2 Age-period-cohort modeling

To disentangle the effects of age, period, and cohort on T2DM trends, Age-period-cohort modeling was applied. This model helps in understanding how different cohorts (birth years), periods (calendar years), and ages influence the rates of T2DM.

#### 2.3.3 Decomposition analysis

This approach quantifies the contributions of population growth, aging, and epidemiological changes to the observed trends in DALYs. This method helps in understanding the relative contributions of these factors to the changing burden of T2DM.

The reasons for our choice of age-period-cohort modeling are presented in **Supplementary Material 2**.

### 2.4 Socio-demographic index

The SDI is a composite indicator that measures a region’s socio-economic status based on income, education, and fertility rates. For this study, countries and regions were categorized into quintiles based on their SDI values: low (SDI <20th percentile), low-middle (20-39th), middle (40-59th), high-middle (60-79th), and high (SDI ≥80th percentile). This categorization allows for the analysis of T2DM trends across different socio-economic contexts.

### 2.5 2044 Diabetes burden projection method

Projections of T2DM burden up to 2044 were conducted using Nordpred prediction model, as implemented in the GBD study. This method utilizes historical data to predict future trends, taking into account current epidemiological and demographic changes.

### 2.6 Uncertainty analysis

To address the inherent variability in DALY rates and model predictions, we implemented a robust uncertainty analysis using bootstrap resampling techniques. This approach helps quantify the uncertainty surrounding our estimates of DALY increases and mortality rates, providing more reliable results for interpretation. We assumed that the DALY rates followed log-normal distributions based on the nature of the data, where values are positive and may exhibit right-skewness. The log-normal assumption is appropriate here as it allows for the modeling of variables that cannot be negative and where the underlying processes that generate the data are multiplicative rather than additive. Specifically, this assumption accommodates the variability often observed in epidemiological data, which can reflect a combination of different risk factors influencing health outcomes. To generate uncertainty intervals (UIs), we applied bootstrap resampling techniques, which involved drawing 1,000 samples with replacement from the original dataset. For each bootstrap sample, we recalculated the DALY and mortality estimates, thereby generating a distribution of estimates. From this distribution, we computed the 2.5th and 97.5th percentiles to establish 95% uncertainty intervals. This method allows us to capture the variability in our estimates due to sampling uncertainty, which is particularly important when working with complex health data. All statistical analyses were conducted using Python version 3.7.3, with results deemed statistically significant at p < 0.05.

Details of all of the above methods can be found in **Supplementary Material 3**.

## 3 Results

### 3.1 Deaths and DALYs of T2DM in 2021

**Figure 1:**
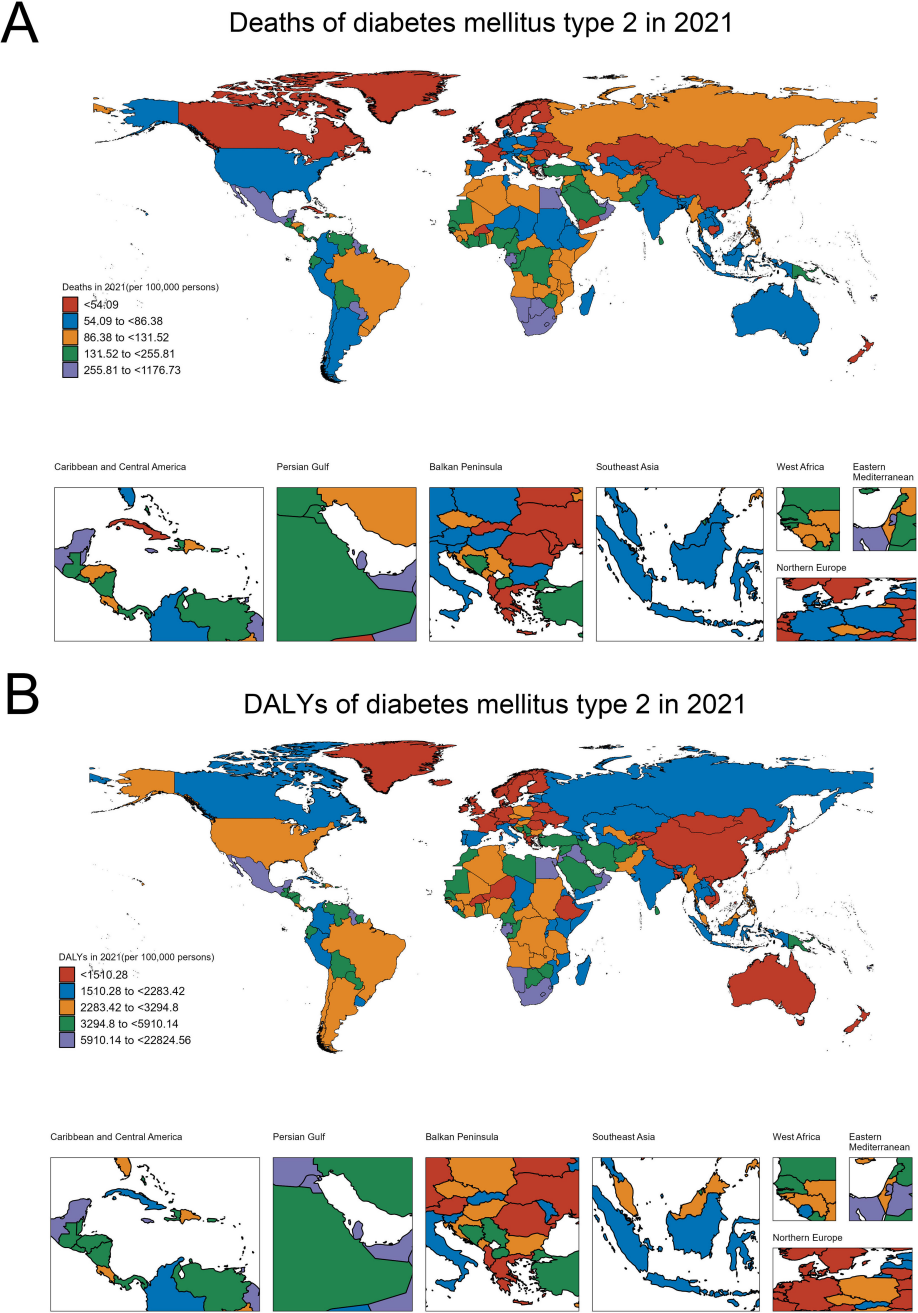
Global burden of T2DM in 2021: Distribution of deaths and DALYs. **(A)** Global distribution of T2DM-related deaths per 100,000 persons. **(B)** Global distribution of T2DM-related DALYs per 100,000 persons.

In 2021, the global distribution of type 2 diabetes-related mortality showed significant regional variations (**Figure 1A**). Countries were classified into five categories based on their mortality rates, ranging from less than 54.09 to as high as 1176.73 deaths per 100,000 people. The lowest mortality rates (<54.09 per 100,000) were found in regions such as East Asia (China, Japan, South Korea), Nordic countries (Sweden, Norway, Finland), and Canada. These areas generally benefit from strong healthcare systems and effective diabetes management programs. Countries with slightly higher, but still relatively low, mortality rates (54.09 to 86.38 per 100,000) included parts of Western Europe (France, Germany), the United States, and several regions in South America (Argentina), Southeast Asia (Malaysia), and Australia. The moderate mortality category (86.38 to 131.52 per 100,000) encompassed regions like Russia, parts of Brazil, and several African countries (Somalia, Kenya, Morocco). Higher mortality rates (131.52 to 255.81 per 100,000) were observed in countries such as Saudi Arabia, Iraq, Pakistan, and Nigeria, while the highest diabetes-related mortality rates (255.81 to 1176.73 per 100,000) were concentrated in South Africa, Egypt, Oman, and Mexico.

Similarly, the distribution of disability-adjusted life years (DALYs) in 2021, which reflects both mortality and morbidity, shows a clear disparity across regions (**Figure 1B**). Countries with the lowest DALYs (<1510.28 per 100,000) included Sweden, Norway, Finland, Western Europe (France, Germany), Australia, and China, which are better equipped to manage the disease and its complications. Moderately low DALYs (1510.28 to 2283.42 per 100,000) were seen in Canada, Russia, Kazakhstan, and India, while moderate DALYs (2283.42 to 3294.8 per 100,000) were found in countries such as Brazil, Argentina, the United States, Algeria, and Myanmar. The category of moderately high DALYs (3294.8 to 5910.14 per 100,000) included Bolivia, Paraguay, Iran, and Saudi Arabia. The highest DALYs (5910.14 to 22,824.56 per 100,000) were concentrated in South Africa, Mexico, Egypt, and Oman, highlighting regions where diabetes is not only a leading cause of death but also contributes heavily to disability and long-term health complications.

### 3.2 Trends in DALYs of T2DM from 1990 to 2021 across SDI levels

**Figure 2:**
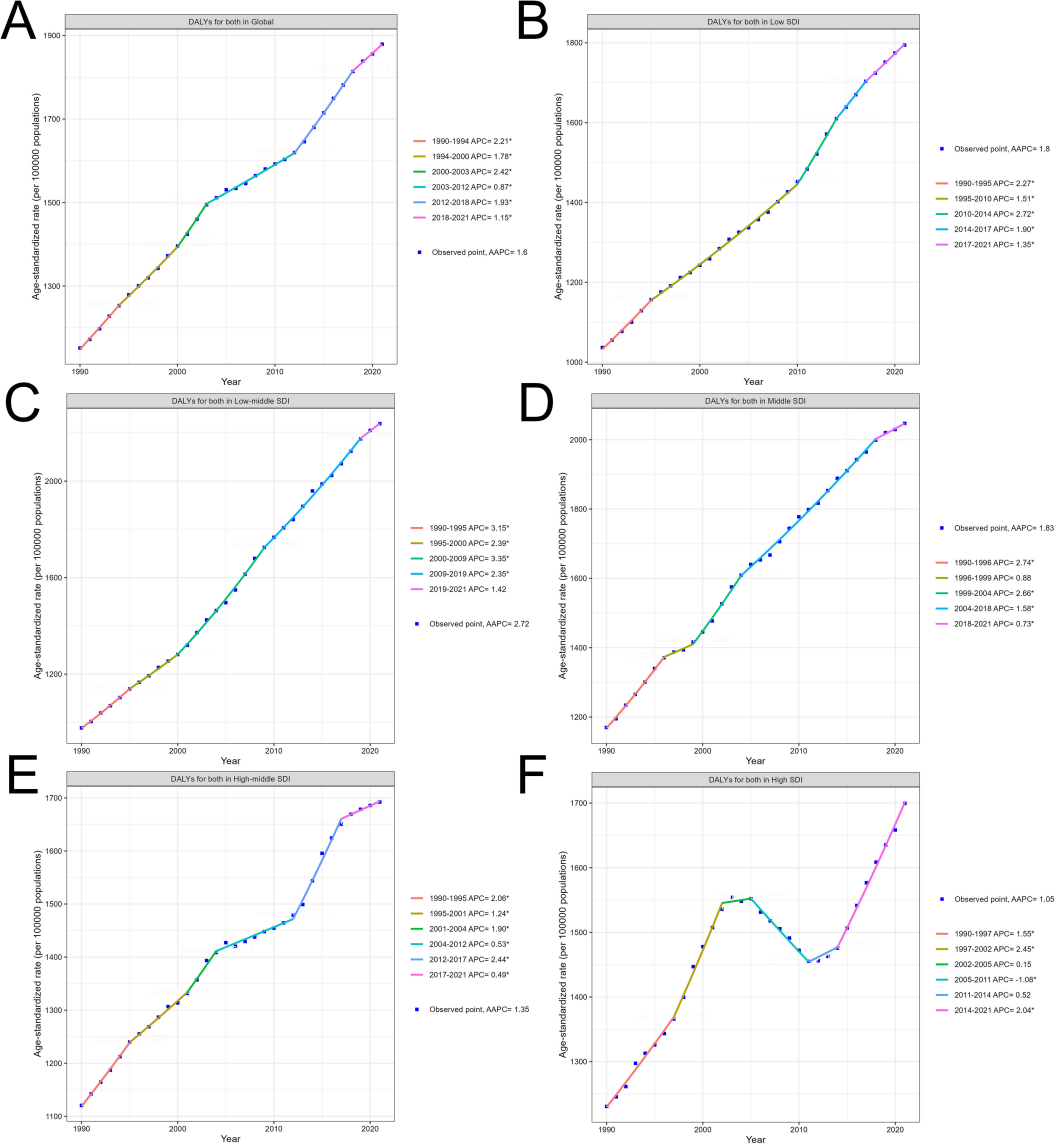
Trends in T2DM burden from 1990 to 2021 by SDI levels: Age-standardized DALYs. **(A)** Global trend of age-standardized DALYs per 100,000 persons from 1990 to 2021. **(B)** Trend of age-standardized DALYs in Low SDI regions from 1990 to 2021. **(C)** Trend of age-standardized DALYs in Low-middle SDI regions from 1990 to 2021. **(D)** Trend of age-standardized DALYs in Middle SDI regions from 1990 to 2021. **(E)** Trend of age-standardized DALYs in High-middle SDI regions from 1990 to 2021. **(F)** Trend of age-standardized DALYs in High SDI regions from 1990 to 2021.

Globally, the burden of DALYs for diabetes mellitus type 2 steadily increased from 1990 to 2021, reflecting broader global health trends. The overall global increase (AAPC 1.6%) demonstrates periods of both acceleration and deceleration, with significant growth observed between 1990-1994 (annual percent change 2.21%) and a slower rise from 2003 to 2012 (annual percent change 0.87%), before picking up again in recent years (annual percent change 1.93% from 2012 to 2018). This general upward trend underscores the expanding global burden of diabetes as a public health issue (**Figure 2A**).

Across SDI regions, the rise in DALYs is marked by varying intensities. Low and low-middle SDI regions showed the steepest increases. In low SDI regions (AAPC 1.8%), a consistent rise in DALYs occurred, with noticeable accelerations during the 2010-2014 period (annual percent change 2.72%), likely reflecting rapid demographic shifts and increased prevalence of risk factors in these areas (**Figure 2B**). Low-middle SDI regions, with the highest AAPC of 2.72%, experienced a significant surge in DALYs from 2000 to 2009 (annual percent change 3.35%), followed by a gradual deceleration. These regions face an escalating diabetes burden driven by rising urbanization, changes in lifestyle, and limited healthcare resources (**Figure 2C**).

Middle SDI regions (AAPC 1.83%) also experienced a notable rise in DALYs, with a strong early growth between 1990 and 1996 (annual percent change 2.74%), followed by more gradual increases in the following decades. This trend reflects the transition of these regions through stages of socioeconomic development, where improvements in healthcare may lag behind rising diabetes risk (**Figure 2D**).

In contrast, high-middle and high SDI regions displayed more stable trends. High-middle SDI regions showed moderate growth (AAPC 1.35%), with brief accelerations such as between 2012 and 2017 (annual percent change 2.44%), followed by slower growth in the subsequent years (**Figure 2E**). High SDI regions exhibited the lowest overall AAPC (1.05%) and more variability over time, including a brief period of decline from 2005 to 2011 (annual percent change -1.08%). However, DALYs in these regions rose again significantly from 2014 to 2021 (annual percent change 2.04%) as lifestyle-related factors outweighed earlier preventive measures (**Figure 2F**). The specific information of AAPC in **Supplementary Material 4**. The specific information of annual percent change in **Supplementary Material 5**. A bar chart of the AAPC data is included in **Supplementary Material 6**.

### 3.3 Net drift and age effect in T2DM across SDI levels

**Figure 3:**
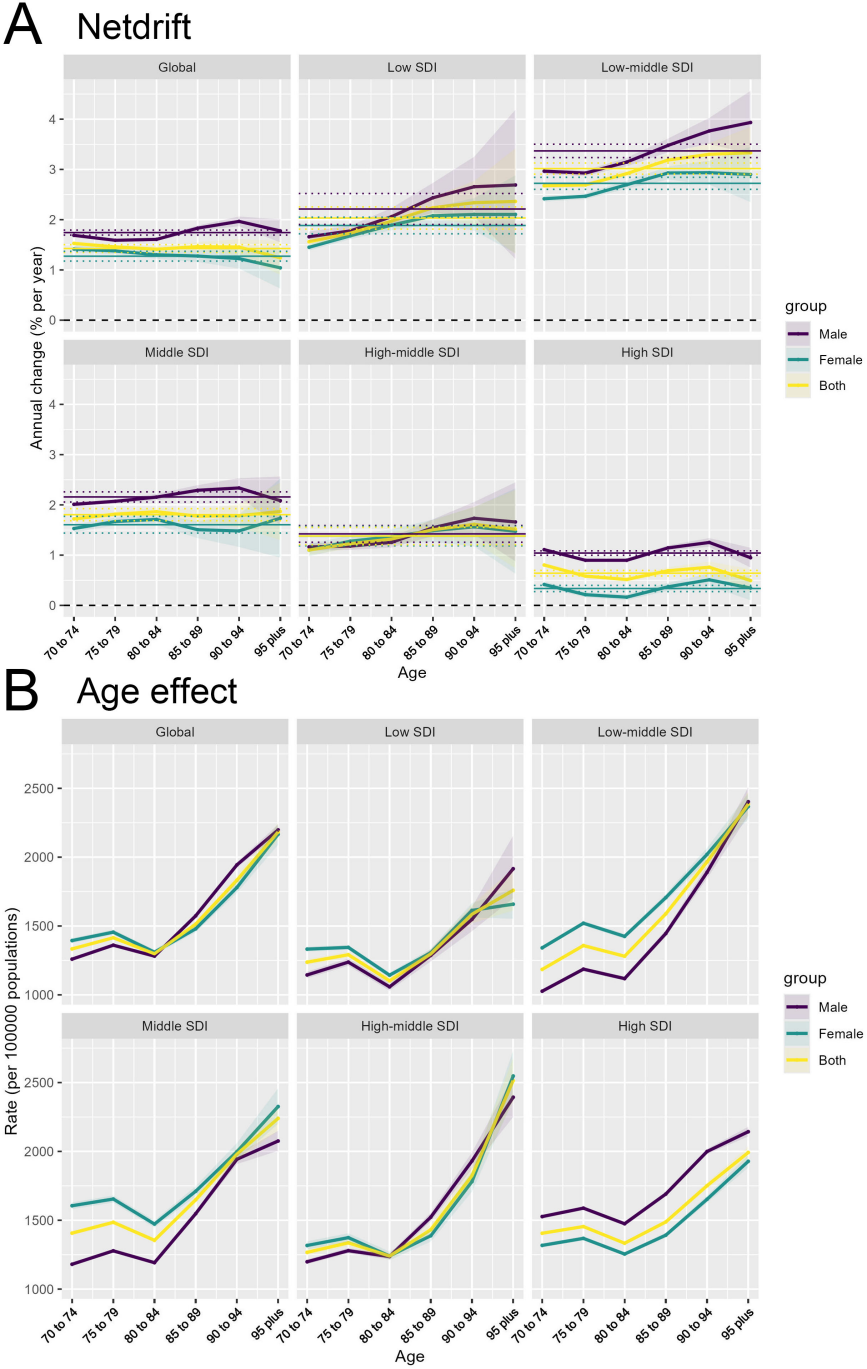
Changes in T2DM incidence across age and SDI levels: Net Drift and Age effects. **(A)** Net drift of annual percentage change in T2DM incidence rates across age groups, shown globally and across different SDI levels for males, females, and both sexes combined. **(B)** Age effect on T2DM incidence rates per 100,000 persons across different age groups, shown globally and across different SDI levels for males, females, and both sexes combined.

In 2021, global trends in T2DM showed notable variations in net drift across different SDI levels and between genders. For males worldwide, the net drift in DALYs related to T2DM was 1.74% (95% CI, 1.69 to 1.79), while for females, it was 1.27% (95% CI, 1.18 to 1.37), indicating that the increase in DALYs was slightly higher among males than females. Among the different SDI levels, males in the low-middle SDI regions exhibited the highest annual increase rate at 3.37% (95% CI, 3.24 to 3.50), whereas males in high SDI regions showed a lower trend with an annual increase of 1.04% (95% CI, 1.00 to 1.09). For females, the low-middle SDI regions also displayed the highest growth rate at 2.72% (95% CI, 2.60 to 2.84), while in high SDI regions, the increase was minimal at 0.34% (95% CI, 0.27 to 0.40). Overall, the combined annual increase rate for both sexes in low-middle SDI regions was 3.02% (95% CI, 2.91 to 3.13), while in high SDI regions, it was 0.64% (95% CI, 0.59 to 0.70) (**Figure 3A**).

### 3.4 Age, period, and cohort effects on T2DM incidence and mortality, 1990-2021

#### 3.4.1 Age effect

The age effect refers to the impact of aging on the risk of developing or dying from T2DM. As observed (**Figure 3B**), both the incidence and mortality rates of T2DM generally increase with age across all SDI regions and globally, but there is a decline in the 75-84 age group. This trend is consistent across genders (male, female, and combined). After age 84, both incidence and mortality rates rise sharply, highlighting the physiological vulnerability of the elderly to the onset of diabetes and its complications.

#### 3.4.2 Cohort effect

**Figure 4:**
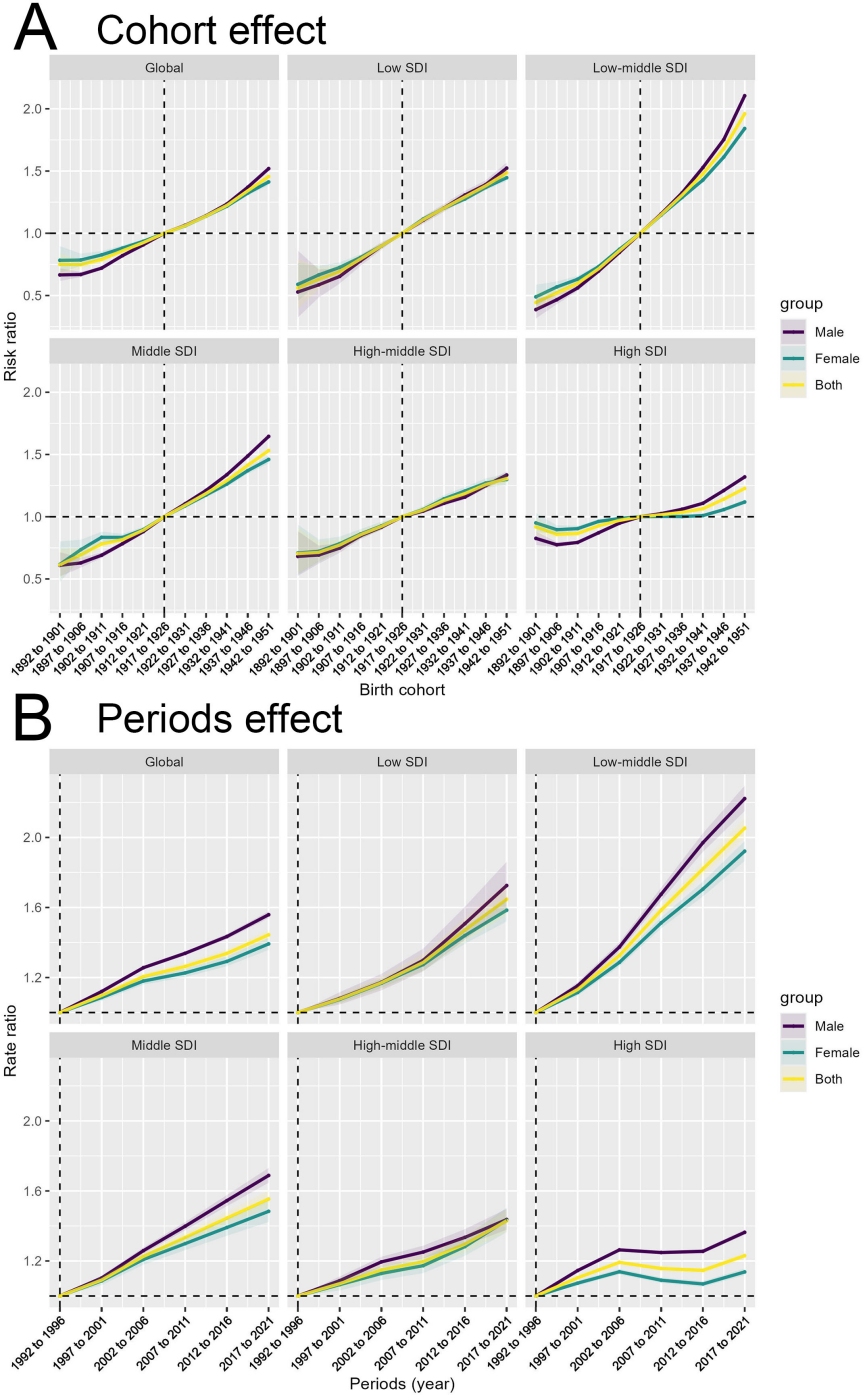
Birth cohort and time period impacts on T2DM incidence across SDI levels. **(A)** Cohort effect on T2DM incidence shown as risk ratios across birth cohorts, globally and across different SDI levels for males, females, and both sexes combined. **(B)** Period effect on T2DM incidence shown as risk ratios across different time periods, globally and across different SDI levels for males, females, and both sexes combined.

The cohort effect (**Figure 4A**) captures the impact of birth cohorts on the incidence and mortality rates of T2DM, reflecting how the risk evolves for individuals born within a specific time frame. We observed that individuals born after 1942-1951 generally showed a higher risk of developing and dying from type 2 diabetes compared to older people born before that. This trend is consistent across all SDI regions, with the most pronounced increase observed in Low-middle SDI regions. This cohort effect may be explained by generational shifts in lifestyle, particularly the transition from traditional diets to more processed and calorie-dense foods, decreased physical activity, and greater exposure to risk factors for obesity and diabetes. For those born later, these environmental shifts occur early in life, contributing to increased lifetime exposure to risk factors, and subsequently, a higher lifetime risk of diabetes. For mortality, the cohort effect likely reflects improvements in healthcare and management of diabetes over time, as older cohorts experienced fewer of these advancements during their earlier years. Despite healthcare improvements, the rising incidence of diabetes in younger cohorts poses a significant challenge for public health systems globally.

#### 3.4.3 Period effect

The period effect (**Figure 4B**) reflects the impact of environmental, medical, and social factors on the incidence and mortality rates of T2DM during specific time periods. The trend in risk ratios from 1990 to 2021 shows a significant increase across all SDI regions, particularly in the Low-middle SDI region. Over time, significant advancements in medical technology, diagnostics, and awareness may have influenced these period trends by improving case detection or altering mortality rates. The consistent rise in risk ratios globally and across all SDI levels indicates that the factors driving the diabetes epidemic are not confined to any particular region or group. However, differences in the magnitude of the rise suggest that high SDI regions may have benefited more from advancements in medical care, reflected by a slower increase in mortality compared to low-SDI regions, where healthcare access remains a significant barrier.

### 3.5 Drivers of the global and regional burden of T2DM, 1990-2021

**Figure 5:**
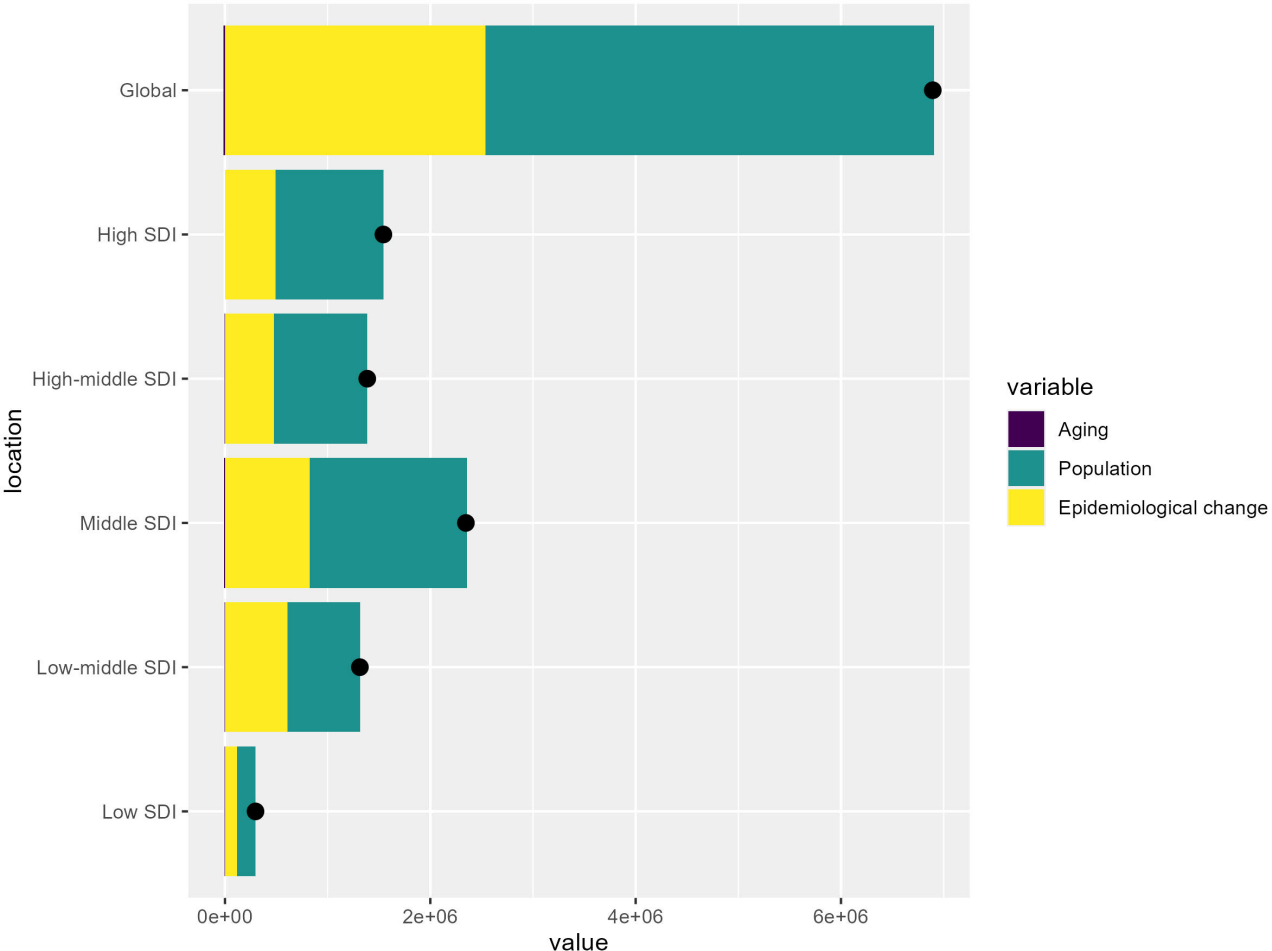
Key drivers of T2DM burden across SDI levels: Aging, population growth, and epidemiological change. The bar chart shows the relative contributions of aging, population growth, and epidemiological change to the overall T2DM burden globally and across different SDI levels.

The global burden of T2DM from 1990 to 2021 is primarily driven by population growth, which is especially significant in younger populations and regions experiencing rapid demographic expansion. The increasing number of people susceptible to diabetes globally reflects the direct impact of population growth on the prevalence of the disease. Additionally, epidemiological changes, such as shifts in lifestyle, marked by greater urbanization, increasing rates of obesity, and more sedentary behaviors, play a crucial role in fueling the diabetes epidemic worldwide. However, aging contributes less significantly to the global T2DM burden, indicating that while an older population is more prone to diabetes, its impact is relatively smaller at a global scale (**Figure 5**).

### 3.6 Predicted rise in T2DM cases and incidence rates from 1990 to 2044

**Figure 6:**
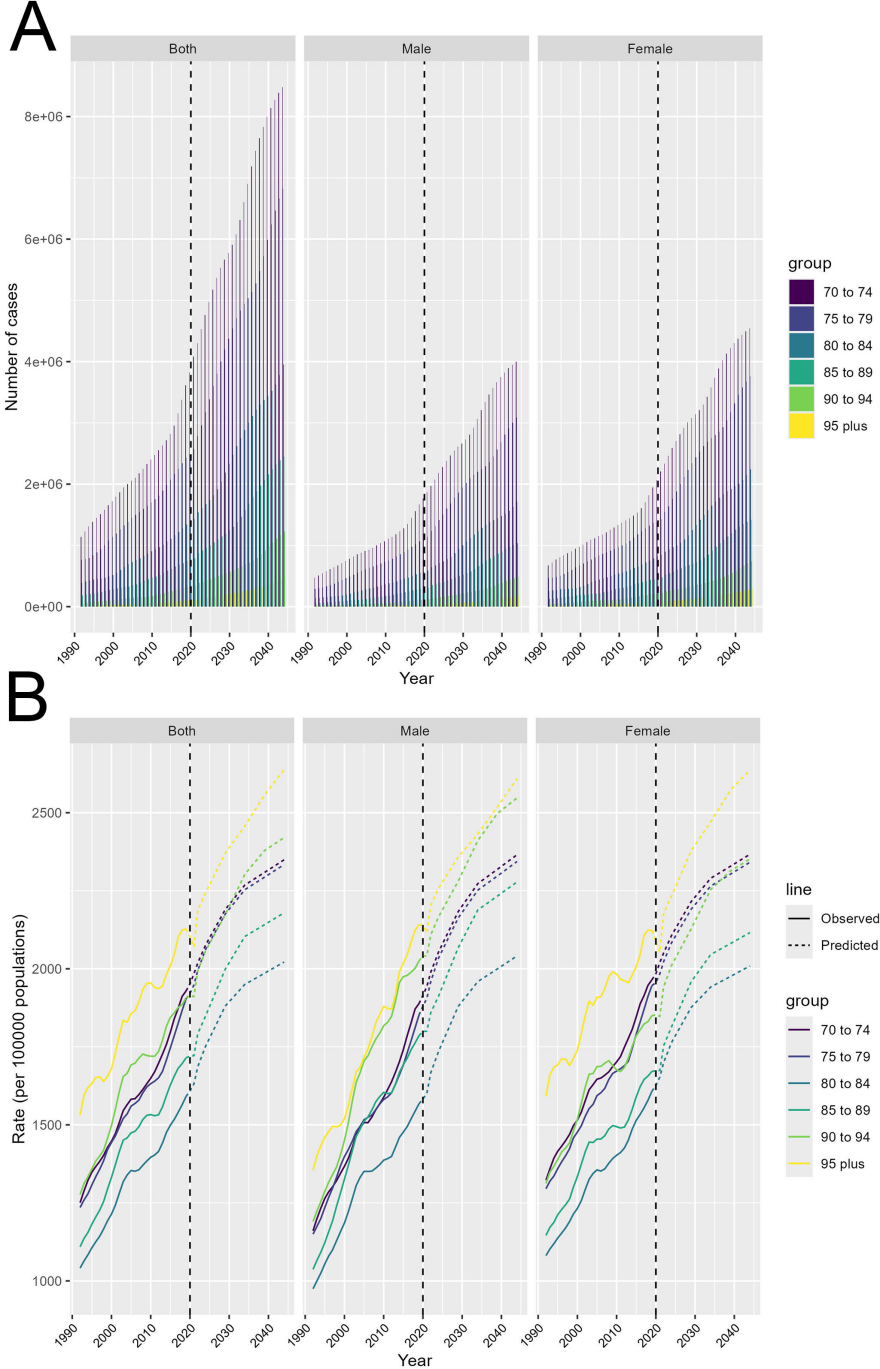
Future projections of T2DM cases and incidence rates by age group from 1990 to 2044. **(A)** Projected number of T2DM cases from 1990 to 2044, shown for both sexes combined, males, and females, stratified by age groups (70-74, 75-79, 80-84, 85-89, 90-94, and 95+ years). **(B)** Projected incidence rates of T2DM per 100,000 persons from 1990 to 2044, shown for both sexes combined, males, and females, stratified by age groups (70-74, 75-79, 80-84, 85-89, 90-94, and 95+ years). Observed (solid lines) and predicted (dashed lines) incidence rates are provided.

Projections for T2DM from 1990 to 2044 show a significant rise in both the number of cases and incidence rates among all age groups over 70. After 2020, the number of T2DM cases is expected to increase sharply, with the most noticeable growth in the 70-74 age group. Although smaller, the number of cases in the elderly population (those over 95) is also predicted to rise substantially. This sharp upward trend indicates that population aging will be a key driver of the future T2DM burden. Both men and women are expected to follow similar trends, although women will be slightly more affected in terms of case numbers (**Figure 6A**). Incidence rates are also expected to increase steadily, with the highest rates projected for the oldest age group (over 95). The continued growth across all age groups suggests that the risk of developing T2DM will increase as the population ages. The projections highlight that healthcare systems must prepare for the significant increase in diabetes cases after 2020, particularly among the elderly (**Figure 6B**). These findings emphasize the urgent need for public health interventions focused on the aging population. Strategies should prioritize prevention, lifestyle modifications, and improved diabetes management to address the rising burden, especially among older adults, whose cases and incidence rates are predicted to increase substantially in the coming decades.

## 4 Discussion

### 4.1 Analysis and implications of study findings

The results of this study provide significant insights into the global and regional trends in T2DM from 1990 to 2021, with projections up to 2044. The increasing burden of T2DM, particularly in low and low-middle SDI regions, highlights the role of population growth and epidemiological changes as key drivers of the disease. These findings align with global health trends, where rapid urbanization, changes in lifestyle, and limited access to healthcare exacerbate the prevalence of diabetes in less developed regions (17, 18). These findings underscore the pressing need for targeted public health interventions and highlight disparities in T2DM management across socio-demographic contexts.

The sharp increase in T2DM incidence and mortality in low and low-middle SDI regions is primarily driven by population growth and urbanization. As these regions undergo economic transitions (19), shifting dietary patterns (towards processed and calorie-dense foods) (20, 21), coupled with reduced physical activity (22), are driving rising obesity rates, a critical risk factor for T2DM (23–25). The lack of robust healthcare systems in these regions exacerbates the challenge, as access to early diagnosis, preventive care, and treatment is often limited (8). Consequently, as demonstrated by the results, T2DM incidence is expected to grow disproportionately in these regions, straining healthcare systems. This emphasizes the urgent need for large-scale public health initiatives that focus on early intervention, education, and improving access to healthcare services.

In high and high-middle SDI regions, the increase in T2DM burden is more closely tied to aging populations. The results of this study confirm that aging is a major driver of the disease burden in these regions, as older age groups are at significantly higher risk for developing T2DM and its complications (26, 27). While healthcare systems in high-SDI regions are generally better equipped to manage chronic conditions like diabetes (28), the continuous rise in cases driven by aging populations points to the need for healthcare systems to evolve, placing a greater emphasis on managing chronic diseases in elderly populations (29, 30). Preventive strategies focusing on healthy aging, physical activity, and controlling risk factors such as obesity and hypertension in older adults should be prioritized moving forward.

The age-period-cohort analysis provides further insights into the dynamics of T2DM across different regions. The age effect, which highlights the increased vulnerability of older populations, is consistent across SDI levels but is more pronounced in high-SDI regions where life expectancy is higher. The period effect, reflecting societal changes over time, underscores how advancements in healthcare and increased awareness have altered T2DM trends, particularly in high-SDI regions where improved diabetes management has helped mitigate mortality. However, the cohort effect is concerning, later birth cohorts, especially in low-SDI regions, show a higher lifetime risk of developing T2DM, driven by early exposure to poor lifestyle factors (31, 32). This suggests that without timely interventions targeting younger populations, the T2DM epidemic will continue to worsen.

In high-SDI regions, where aging is the dominant factor, healthcare systems must prioritize managing chronic diseases in older populations. Integrating diabetes management into broader aging and health frameworks could help reduce the long-term T2DM burden. Policymakers and healthcare providers should focus on preventive measures that encourage healthy aging, including interventions aimed at controlling obesity, hypertension, and maintaining physical activity levels in older adults. In low- and middle-SDI regions, where population growth and epidemiological shifts drive the rise of T2DM, multifaceted public health strategies are essential. Key interventions should include promoting healthier diets through public health campaigns that encourage the consumption of whole foods while reducing processed foods high in sugar and unhealthy fats (33). Additionally, increasing physical activity is vital (34, 35); local governments can invest in safe walking and biking paths, while schools implement mandatory physical education and after-school sports programs. Community-based exercise initiatives, like walking clubs, can foster social support. Finally, raising awareness about diabetes risks through comprehensive educational campaigns using various media platforms, along with tailored materials for different literacy levels, can ensure that vital information reaches all populations (36).

### 4.2 A considerations and broader context

#### 4.2.1 Socioeconomic diversity and limitations of the SDI grouping

While this study utilizes the SDI as an effective tool to categorize regions based on socio-economic factors, it is important to recognize its limitations in capturing the full complexity of socioeconomic diversity within countries. Large, heterogeneous nations like China, India, and United States exhibit significant disparities in healthcare access, public health infrastructure, and economic conditions across different regions (37, 38). These variations can result in notable differences in the incidence and management of T2DM. For instance, rural areas in these countries often face higher diabetes complications and poorer healthcare access compared to urban areas (39–41). As such, while SDI serves as a useful metric for broad analysis, a more granular approach (such as sub-national studies) would provide deeper insights into these within-country disparities and better inform targeted public health interventions.

#### 4.2.2 Uncertainty of future projections and role of technological advancements

The projections provided in this study are based on historical data and trends, yet it is crucial to consider the uncertainties inherent in these forecasts. Future advancements in diabetes treatment and management, healthcare reforms, and global public health initiatives may significantly alter the predicted trajectory of T2DM. For example, innovations such as improved pharmacological therapies (42) and novel prevention strategies (43) could potentially reduce the global burden of diabetes. Additionally, large-scale public health initiatives aimed at improving healthcare infrastructure in low- and middle-SDI regions could mitigate the projected rise in diabetes cases (44). Therefore, these forecasts should be interpreted with caution, and policymakers should consider them as a flexible framework rather than a fixed outcome.

#### 4.2.3 The influence of genetics and environmental exposures

In addition to the epidemiological and demographic factors emphasized in this study, other critical contributors to T2DM, such as genetics and environmental exposures, also warrant attention. Genetic predispositions, including insulin resistance and beta-cell dysfunction, can significantly impact an individual’s risk of developing diabetes (45). Moreover, environmental factors, such as exposure to pollutants (46), endocrine-disrupting chemicals (47), and socio-environmental stressors (31), may further exacerbate diabetes risk, particularly in vulnerable populations. Although these aspects were not the primary focus of this analysis, acknowledging their influence is essential for a more comprehensive understanding of global T2DM trends. Future research should explore these factors in greater depth to better elucidate their roles in diabetes prevalence and outcomes.

Overall, the study highlights the importance of early prevention and region-specific strategies to mitigate the future burden of T2DM. By addressing the distinct drivers of T2DM in different SDI contexts, policymakers and public health professionals can develop more effective interventions, helping to curb the rising global diabetes epidemic.

## 5 Conclusion

In summary, this study highlights the global and regional trends in T2DM from 1990 to 2021, with projections showing continued case and incidence increases through 2044. Population growth, aging, and epidemiological changes are the primary drivers of this trend, with low- and middle-SDI regions experiencing the most significant increases. While high-SDI regions also face growing burdens, particularly due to aging populations, their more robust healthcare systems provide some mitigation. These findings underscore the urgent need for region-specific interventions targeting lifestyle risk factors and improving healthcare access to curb the rising T2DM burden. Addressing these challenges will be critical to reducing future morbidity and mortality associated with T2DM globally.

## Data Availability

The original contributions presented in the study are included in the article/**Supplementary Material**. Further inquiries can be directed to the corresponding authors.

## Supplementary material

Supplementary Material 1 More information on the validity and reliability of the GBD 2021 dataset is provided.

Supplementary Material 2 The reasons for our choice of age-period-cohort modeling are presented.

Supplementary Material 4 The specific information of AAPC.

Supplementary Material 5 The specific information of annual percent change.

Supplementary Material 6 A bar chart of the AAPC data is included.
