# Supplementary material for: Global burden of type 2 diabetes mellitus from 1990 to 2021, with projections of prevalence to 2044: a systematic analysis across SDI levels for the global burden of disease study 2021
Source: Front Endocrinol (Lausanne). 2024 Nov 8;15:1501690. doi: 10.3389/fendo.2024.1501690 (PMC11581865; doi:10.3389/fendo.2024.1501690)
Supplement: Supplementary Material 3 — Details of all of the above methods can be found. [file DataSheet3.docx]

**Validity of the GBD 2021 dataset**

**Comprehensive scope:** The GBD 2021 dataset encompasses a wide array of health metrics, particularly concerning Type 2 Diabetes Mellitus (T2DM) mortality and disability-adjusted life years (DALYs) across different regions and demographics. This breadth allows for an in-depth analysis of the global burden of T2DM, facilitating the identification of trends and disparities that are crucial for public health policy formulation.

**Rigorous methodology:** The GBD employs a systematic approach to data collection and estimation, utilizing various methodologies, including meta-analyses, vital registration data, and health surveys. This rigorous methodology enhances the reliability of the findings, allowing us to draw robust conclusions from the data. It is noteworthy that GBD data are continually updated to reflect the latest research and statistical techniques, ensuring that the findings are based on the most accurate and comprehensive information available.

**Temporal trends analysis:** The dataset spans several decades, providing a longitudinal perspective that is essential for assessing trends in T2DM incidence and mortality. This temporal aspect is particularly valuable for understanding how demographic and epidemiological factors have evolved over time. The ability to analyze data from 1990 to 2021 enables us to observe changes in health outcomes and the impact of various interventions over time.

**SDI framework:** By incorporating the SDI, which combines income, education, and fertility rates, the GBD dataset allows for the exploration of T2DM trends in relation to socio-economic factors. This framework is particularly relevant for analyzing the rising incidence of T2DM in low and middle-income countries, where socio-economic disparities significantly influence health outcomes. By categorizing countries into different SDI levels, we can better understand how these disparities affect T2DM burden across regions.

**Public accessibility and transparency:** The GBD data are publicly available, enabling reproducibility and transparency in research. This accessibility allows for independent verification of our findings, thereby strengthening the overall validity of our study. Researchers and policymakers can utilize these data for their analyses, contributing to a more extensive understanding of global health trends.

**Addressing limitations**

We acknowledge that the GBD 2021 dataset has certain limitations, particularly regarding disparities related to race and socio-economic status, especially in regions with limited healthcare infrastructure where data quality might be compromised. To address these concerns, we employed the following strategies:

**Data cleaning and validation:** We undertook comprehensive data cleaning processes to ensure the integrity of the dataset, addressing any inconsistencies and outliers. This process included verifying the data against multiple sources where available, ensuring that our analyses are based on the highest quality data.

**Acknowledgment of disparities:** We explicitly discussed the limitations related to racial and ethnic disparities within the GBD dataset. We recognize that regions with limited healthcare infrastructure may experience challenges in data collection and reporting. Future research is needed to capture these nuances more effectively and to ensure that public health interventions are tailored to address these disparities.

**Stratification of analysis:** We conducted a stratified analysis based on SDI levels and age groups, which provides a clearer understanding of how various populations are affected by T2DM. This stratification allows us to identify specific at-risk groups and understand the disparities in disease burden across different demographics.

**Sensitivity analyses:** To assess the robustness of our findings, we performed sensitivity analyses to evaluate the potential impact of data limitations on our results. This analysis helps ensure that our conclusions are reliable and not overly influenced by any specific data constraints.
