# Supplementary material for: Global burden of type 2 diabetes mellitus from 1990 to 2021, with projections of prevalence to 2044: a systematic analysis across SDI levels for the global burden of disease study 2021
Source: Front Endocrinol (Lausanne). 2024 Nov 8;15:1501690. doi: 10.3389/fendo.2024.1501690 (PMC11581865; doi:10.3389/fendo.2024.1501690)
Supplement: Supplementary Material 4 — The specific information of AAPC. [file DataSheet4.docx]

**Justification for using APC modeling**

**Ability to decompose temporal trends:**

APC modeling is particularly well-suited for studies like ours that aim to disentangle the distinct effects of age, period, and cohort on disease trends. The APC model allows us to:

Assess age effects, which represent the changes in disease risk as individuals grow older.

Examine period effects, reflecting changes in external factors (e.g., medical advances, public health policies) that affect all age groups simultaneously.

Analyze cohort effects, which capture the unique influences of birth cohorts due to environmental, social, or behavioral factors during early life.

By separating these three components, APC models provide a more granular understanding of how T2DM burden has evolved over time and across different demographics. Alternative approaches, such as GAMs, do not allow for such explicit separation of these three effects, which is essential for our study's objective.

**Flexibility in modeling non-linear trends:**

While GAMs are powerful for capturing non-linear relationships, APC modeling offers specific advantages for epidemiological studies where non-linearity can arise from the complex interactions of age, period, and cohort factors. APC models can flexibly accommodate non-linear trends by allowing for distinct contributions of each factor. This is particularly important for our study, where T2DM trends are influenced by aging populations, shifts in healthcare practices, and socio-economic changes over time.

**Robustness for longitudinal data:**

APC models are well-established for longitudinal epidemiological data like those from the Global Burden of Disease (GBD) study, which spans several decades. APC models offer a robust framework for capturing the temporal patterns that emerge across different generations and time periods, making it ideal for projecting future trends in T2DM burden. In contrast, methods like GAMs or other time-series techniques are often better suited for short-term trend analysis or when the primary interest lies in predicting outcomes without the need to disentangle cohort-specific effects.

**Interpretability and public health relevance:**

One of the strengths of the APC model is its interpretability in public health research. By breaking down the impact of age, period, and cohort, the model provides results that are directly relevant for designing targeted interventions. For example, cohort effects can highlight vulnerable populations based on their birth years, informing public health strategies that target specific age groups or generational cohorts. This level of insight is critical for guiding future public health interventions in T2DM management, making the APC model the most appropriate choice for our research question.

**Previous successful applications:**

APC modeling has been successfully used in numerous high-impact studies analyzing chronic disease trends, including those related to cancer, cardiovascular disease, and diabetes. Its widespread application and proven effectiveness in epidemiological research further support its use in our study. We have built upon this established methodology to ensure the robustness of our findings.

**Limitations of alternative methods**

While alternative methods like GAMs or other time-series models are valuable for different types of analyses, they were not selected for the following reasons:

**GAMs:** Although GAMs are flexible and useful for smoothing non-linear trends, they do not offer the ability to explicitly separate the effects of age, period, and cohort, which is a central aim of our study.

**Time-series techniques:** While effective for forecasting, time-series models are less suited for disentangling age, period, and cohort effects, which are critical for understanding the long-term epidemiological dynamics of T2DM.
