# Supplementary material for: Global burden of type 2 diabetes mellitus from 1990 to 2021, with projections of prevalence to 2044: a systematic analysis across SDI levels for the global burden of disease study 2021
Source: Front Endocrinol (Lausanne). 2024 Nov 8;15:1501690. doi: 10.3389/fendo.2024.1501690 (PMC11581865; doi:10.3389/fendo.2024.1501690)
Supplement: Supplementary Material 5 — The specific information of annual percent change. [file DataSheet5.docx]

**Supplementary Methods**

**1.Joinpoint regression analysis**

Time trend analysis plays a crucial role in epidemiological research, yet traditional regression models often overlook local variations by only assessing overall trends in disease distribution across the entire study period. In 1998, Kim et al. introduced the Joinpoint regression model to address this limitation. This model offers a segmented approach to regression, dividing the time period into various segments through multiple Joinpoints. Each segment is then analyzed separately, allowing for a more nuanced understanding of disease trends within different intervals of the study period(1). Developed by the Division of Cancer Control and Population Sciences at the National Cancer Institute in the U.S., the Joinpoint regression model has become a valuable tool for studying trends in disease incidence and mortality rates.

**(I) Model Introduction**

The Joinpoint regression model offers two variations: the linear model (y = xb) and the logarithmic linear model (ln y = xb). The linear model is typically preferred when the dependent variable is normally distributed (or approximately normal) and the sample size is sufficiently large (usually over 100). This model is well-suited for continuous variables like height and weight. On the other hand, when the dependent variable adheres to an exponential or Poisson distribution, the logarithmic linear model is more appropriate. This is often the case for epidemiological data related to population metrics, such as incidence rates or case numbers. For instance, when analyzing trends in the incidence, prevalence, mortality rates, and DALYs for thalassemia using population-based data, the logarithmic linear model is typically employed.

**(II) Modeling Method**

The default modeling approach used by Joinpoint is the grid search method (GSM). GSM divides the study data into a grid, with each intersection representing a potential scenario(1). For each scenario within specified intervals, the method calculates performance metrics for the corresponding equations using a fixed step size to identify the optimal function. Essentially, the Joinpoint model leverages GSM to evaluate all potential Joinpoints (i.e., points where segments meet) and computes the sum of squares errors (SSE) and mean squared errors (MSE) for each possibility. It then selects the grid point with the smallest MSE as the Joinpoint and uses it to fit the parameters of the equation, such as β_0_, β_1_, δ_1_, ..., δ_k_, based on the identified Joinpoints and the respective interval functions(2).

**(III) Model Optimization**

In Joinpoint software, the default method for model optimization is the Monte Carlo permutation test. Before starting the modeling process, the range for the number of Joinpoints, k, needs to be defined as k ∈ (MIN, MAX), where MIN is typically set to 0, representing the minimum number of Joinpoints, and MAX represents the maximum allowed number of Joinpoints. The test evaluates the null hypothesis (H_0_: the number of Joinpoints is k = k_a_) against the alternative hypothesis (H_1_: the number of Joinpoints is k = k_b_). The test begins with k_a_=MIN and k_b_=MAX. If H_0_ is rejected, the value of k is incremented to k_a_ + 1 for further testing. If H_0_ is not rejected, k is reduced to k_b_ - 1. This process continues until ka equals k_b_, indicating that the value of k = k_a_ = k_b_ is the optimal number of Joinpoints selected by the permutation test, resulting in the best-fitting model(3).

**(IV) Index Calculation**

In the Joinpoint model, the key outcome indicators are the Annual Percent Change (APC) and Average Annual Percent Change (AAPC), both of which are typically accompanied by their 95% confidence intervals (CI). APC measures the yearly percentage change of the dependent variable on average. For instance, in a logarithmic linear model expressed as ln (y) =β_0_ + β_1_ x, where y is the incidence rate and x represents the year, the APC can be calculated from the fitted model using the formula:


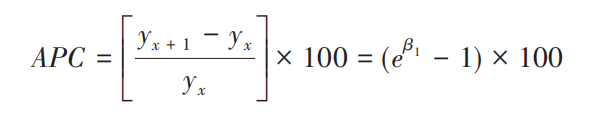


The lower and upper limits of the 100(1-α) % confidence interval are respectively:


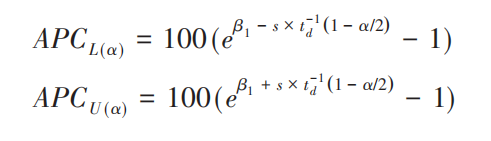


In the given formula, β_1_ represents the regression coefficient, s denotes the standard error of β_1_​, d refers to the degrees of freedom, and t_d_(q) is the critical value corresponding to the qth percentile of the t-distribution with d degrees of freedom (for example, 95%).

The APC is used to assess the trend within each independent interval of a segmented function, or to analyze the overall trend if there are no joinpoints. However, when evaluating the overall average trend across multiple segments, the AAPC is employed. The calculation of AAPC involves a weighted average of the regression coefficients from each segment, with the weights based on the width w of each segment interval. Its formula is as follows:


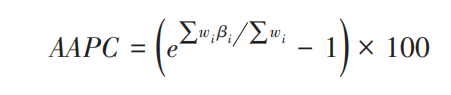


The lower and upper limits of the 100(1-α) % confidence interval are respectively:


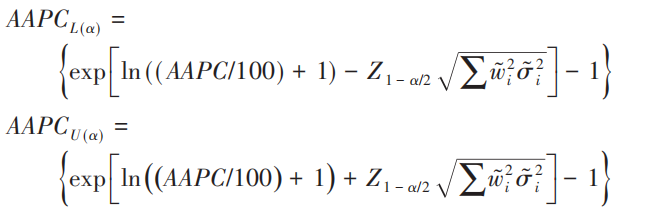


In the given equation, w_i_ is defined as the span, or duration, of each segment function interval, signifying the total years encompassed within each interval. The symbol β_i_ represents the regression coefficient associated with each respective interval, while σ^2^i indicates the variance of β_i_. Furthermore, Z_α_ corresponds to the value at the α percentile within the normal distribution.

**(V) Software Download**

We accessed the National Cancer Institute's website to download the Joinpoint software (available at https://surveillance.cancer.gov/Joinpoint/download). After registering and providing the necessary application details, we successfully obtained the software. For referencing purposes, the software is cited as follows: "Joinpoint Regression Program, Version 4.9.1.0 - April 2022; Statistical Methodology and Applications Branch, Surveillance Research Program, National Cancer Institute."

**2.Age-period-cohort modelling analysis**

For the GBD 2021, mortality estimates for T2DM along with population data from various countries and regions were utilized as input data for the APC model. This model was employed to determine the overall temporal trends in mortality, quantified as the annual percentage change in mortality, or the net drift, expressed in percentage per year. The net drift comprises two main components: the trend attributable to calendar time and the trend attributable to successive cohorts. The APC model also provided estimates of the temporal trends in mortality within specific age groups, denoted as the annual percentage change of age-specific mortality or the local drift, reflecting the influence of birth cohort effects(4). A change in mortality of ±1% per year is deemed significant(4) correlating to changes of approximately ±10%, ±18%, and ±26% in the mortality rate over 10, 20, and 30 years, respectively. The significance of these annual percentage changes was evaluated using a Wald chi-squared test(4). Additionally, the APC model's outputs included fitted longitudinal age-specific rates for the referent cohort, adjusted for period deviations to showcase age-associated natural trends (i.e., age effects), and period (cohort) relative risks of mortality for each period (cohort) to indicate period (cohort) effects(4). The relative risk is calculated as the ratio of age-specific rates in each period (cohort) compared to a reference period (cohort). Both the period (cohort) rate ratio curves encompass the full value of the net drift. The selection of the referent period (cohort) is arbitrary and does not influence the interpretation of the results. All statistical tests were conducted as two-sided with a significance level set at p < 0.05. The analysis was performed using R software, version 3.6.3(5).

**3.Decomposition analysis**

We initially applied the decomposition methodology developed by Das Gupta(6-8) to break down the DALYs associated with T2DM into components attributable to changes in the population age structure, population growth, and epidemiologic shifts. The calculation of DALYs for each location was based on the specific formula outlined below:

DALY _ay, py, ey_ = $\sum_{i=1}^{20} ($a _i, y_ * p _y_ * e _i, y_)

Among them, DALY _ay, py, ey_ denotes DALY based on age structure, population, and DALY rate factors for a particular year y; a _i_ _y_ represents the proportion of the population in age group i out of the 20 age groups in given year y; p _y_ denotes the total population in given year y; and e _i, y_ denotes DALYs rate given age category i in year y.

**4. Nordpred prediction model**

The Nordpred prediction model, derived from the APC framework, effectively forecasts future trends in DALYs. This model takes into account the interplay between time series data and demographic factors, such as shifts in population structure, disease patterns, and generational effects. To project trends, the Nordpred APC model was employed to estimate DALYs from 2022 to 2044. The Nordpred analysis is conducted in five-year intervals of age, period, and cohort, serving as the foundation for projecting trend data for each period. It utilizes a log-linear age-period-cohort model designed to predict the number or rate of new cases, which helps to moderate exponential growth and constrain linear trend projections to align with recent trends, demonstrating effectiveness in forecasting future burden trends. The model extrapolates from the most recent five years of observed data (three or four years, depending on data availability) using a power function to temper growth. Subsequently, the linear trend from the previous decade is adjusted for the second, third, and fourth prediction periods, either diminishing or enhancing it by 25%, 50%, and 75%, respectively. The final predictions for DALYs in 2044 are calculated by averaging the projected incidence rates for the last two prediction periods, centered on the year 2044(9, 10). The Nordpred software package, implemented in R language (version 4.3.1), integrates dynamic changes in incidence rates and population structure.

**References:**

1. Kim HJ, Fay MP, Feuer EJ, Midthune DN. Permutation tests for joinpoint regression with applications to cancer rates. Stat Med. 2000;19(3):335-51.

2. Siegel RL, Miller KD, Fuchs HE, Jemal AJCCJC. Cancer statistics, 2021. 2021;71(1):7-33.

3. Yang JJ, Trucco EM, Buu A. A hybrid method of the sequential Monte Carlo and the Edgeworth expansion for computation of very small p-values in permutation tests. Stat Methods Med Res. 2019;28(10-11):2937-51.

4. Rosenberg PS, Check DP, Anderson WF. A web tool for age-period-cohort analysis of cancer incidence and mortality rates. Cancer epidemiology, biomarkers & prevention : a publication of the American Association for Cancer Research, cosponsored by the American Society of Preventive Oncology. 2014;23(11):2296-302.

5. %J RCT. A language and environment for statistical computing. 2021.

6. P. DG. Standardization and decomposition of rates: a user’s manual, Pages 19-361993.

7. Das Gupta P. Standardization and decomposition of rates from cross-classified data. Genus. 1994;50(3-4):171-96.

8. Chevan A, Sutherland M. Revisiting Das Gupta: refinement and extension of standardization and decomposition. Demography. 2009;46(3):429-49.

9. Arnold M, Park JY, Camargo MC, Lunet N, Forman D, Soerjomataram I. Is gastric cancer becoming a rare disease? A global assessment of predicted incidence trends to 2035. Gut. 2020;69(5):823-9.

10. Jürgens V, Ess S, Cerny T, Vounatsou P. A Bayesian generalized age-period-cohort power model for cancer projections. Statistics in medicine. 2014;33(26):4627-36.
