# Supplementary figures and images for: Global burden of type 2 diabetes mellitus from 1990 to 2021, with projections of prevalence to 2044: a systematic analysis across SDI levels for the global burden of disease study 2021
Source: Front Endocrinol (Lausanne). 2024 Nov 8;15:1501690. doi: 10.3389/fendo.2024.1501690 (PMC11581865; doi:10.3389/fendo.2024.1501690)

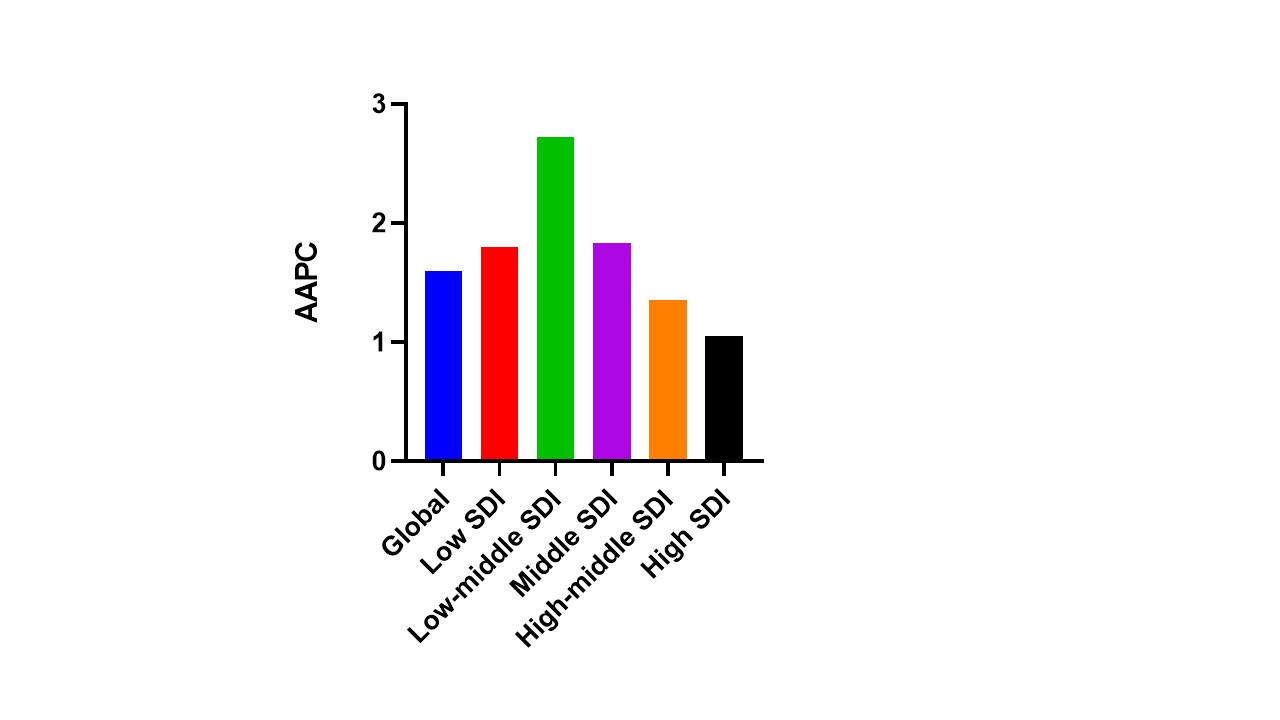

Supplement: Supplementary Material 6 — A bar chart of the AAPC data is included. [file Image1.jpeg]
